# Transduction of rat pancreatic islets with pseudotyped adeno-associated virus vectors

**DOI:** 10.1186/1743-422X-6-61

**Published:** 2009-05-18

**Authors:** Anthony T Craig, Oksana Gavrilova, Nancy K Dwyer, William Jou, Stephanie Pack, Eric Liu, Klaus Pechhold, Michael Schmidt, Victor J McAlister, John A Chiorini, E Joan Blanchette-Mackie, David M Harlan, Roland A Owens

**Affiliations:** 1 Laboratory of Molecular and Cellular Biology, National Institute of Diabetes and Digestive and Kidney Diseases, National Institutes of Health, Department of Health and Human Services, Bethesda, Maryland 20892, USA; 2 Department of Genetics and Human Genetics, Howard University Graduate School, Washington, D.C. 20059, USA; 3 Mouse Metabolism Core Laboratory, National Institute of Diabetes and Digestive and Kidney Diseases, National Institutes of Health, Department of Health and Human Services, Bethesda, Maryland 20892, USA; 4 Laboratory of Cell Biochemistry and Biology, National Institute of Diabetes and Digestive and Kidney Diseases, National Institutes of Health, Department of Health and Human Services, Bethesda, Maryland 20892, USA; 5 Diabetes Branch, National Institute of Diabetes and Digestive and Kidney Diseases, National Institutes of Health, Department of Health and Human Services, Bethesda, Maryland 20892, USA; 6 Molecular Physiology and Therapeutics Branch, National Institute of Dental and Craniofacial Research, National Institutes of Health, Department of Health and Human Services, Bethesda, Maryland 20892, USA

## Abstract

**Background:**

Pancreatic islet transplantation is a promising treatment for type I diabetes mellitus, but current immunosuppressive strategies do not consistently provide long-term survival of transplanted islets. We are therefore investigating the use of adeno-associated viruses (AAVs) as gene therapy vectors to transduce rat islets with immunosuppressive genes prior to transplantation into diabetic mice.

**Results:**

We compared the transduction efficiency of AAV2 vectors with an AAV2 capsid (AAV2/2) to AAV2 vectors pseudotyped with AAV5 (AAV2/5), AAV8 (AAV2/8) or bovine adeno-associated virus (BAAV) capsids, or an AAV2 capsid with an insertion of the low density lipoprotein receptor ligand from apolipoprotein E (AAV2apoE), on cultured islets, in the presence of helper adenovirus infection to speed expression of a GFP transgene. Confocal microscopy and flow cytometry were used. The AAV2/5 vector was superior to AAV2/2 and AAV2/8 in rat islets. Flow cytometry indicated AAV2/5-mediated gene expression in approximately 9% of rat islet cells and almost 12% of insulin-positive cells. The AAV2/8 vector had a higher dependence on the helper virus multiplicity of infection than the AAV 2/5 vector. In addition, the BAAV and AAV2apoE vectors were superior to AAV2/2 for transducing rat islets. Rat islets (300 per mouse) transduced with an AAV2/5 vector harboring the immunosuppressive transgene, *tgfβ1*, retain the ability to correct hyperglycemia when transplanted into immune-deficient diabetic mice.

**Conclusion:**

AAV2/5 vectors may therefore be useful for pre-treating donor islets prior to transplantation.

## Background

Type I diabetes mellitus (Type I DM) is an autoimmune disorder that destroys pancreatic β-cells in the islets of Langerhans, causing severe insulin deficiency and hyperglycemia. Treatment options include islet transplantation, but consistent Type I DM correction with this approach has been elusive, partly due to side-effects of required immunosuppressive drugs [1-3]. Expression of immunosuppressive genes within islets may provide local protection and reduce the need for immunosuppressive drugs. Genes, including those encoding CTLA4Ig [4-6], soluble Fas ligand [6], and transforming growth factor β1 (TGF-β1) [4,7-9] have been transferred to islets in animal transplantation models. One problem is the gene delivery system. Adenoviral vectors provide only transient gene expression, and there are safety questions with retroviral and lentiviral vectors.

Vectors based on adeno-associated viruses (AAVs) have been studied for their abilities to transduce mouse and human islets, since such vectors may overcome the safety and efficacy concerns. The best studied AAV is AAV2, a naturally defective human parvovirus with a 4.7 kb genome [10]. To replicate, AAV2 usually requires a helper virus, such as an adenovirus or herpesvirus [11]. AAV2 has not been associated with a human disease.

There are conflicting reports regarding the ability of AAV2 vectors with an AAV2 capsid (AAV2/2) to transduce efficiently mouse or human islets [12-16]. The abilities of AAV2 vectors pseudotyped with the capsids of AAV1 (AAV2/1) [13,16,17], AAV5 (AAV2/5) [12,13,15,16], and AAV8 (AAV2/8) [14,15,17] to transduce murine and human islets have also been investigated. Another approach to enhance islet transduction is the insertion of the low density lipoprotein receptor ligand from apolipoprotein E into AAV2 capsid proteins (AAV2apoE) [13].

Another roadblock to islet transplantation is the shortage of human donors. Xenotransplantation is being explored to overcome this obstacle [7,8,18] and rat to mouse transfer of islets provides a model system. The relative ease of harvesting large numbers of rat islets, versus murine islets, also makes the use of rat islets desirable. Therefore, we examined the transduction of rat islets by pseudotyped AAV vectors.

## Results

### Transduction of Rat Islets with AAV Vectors

To determine which AAV capsids more efficiently mediate transduction, we transduced rat islets with AAV vectors and simultaneously infected them with Ad5, because islets can only survive for 1 week in tissue culture, and co-infection with Ad5 results in less time before AAV vector transgene expression. As a transduction marker, the eGFP gene, modified to confer nuclear localization (nls-eGFP), was included in the vectors.

**Figure 1:**
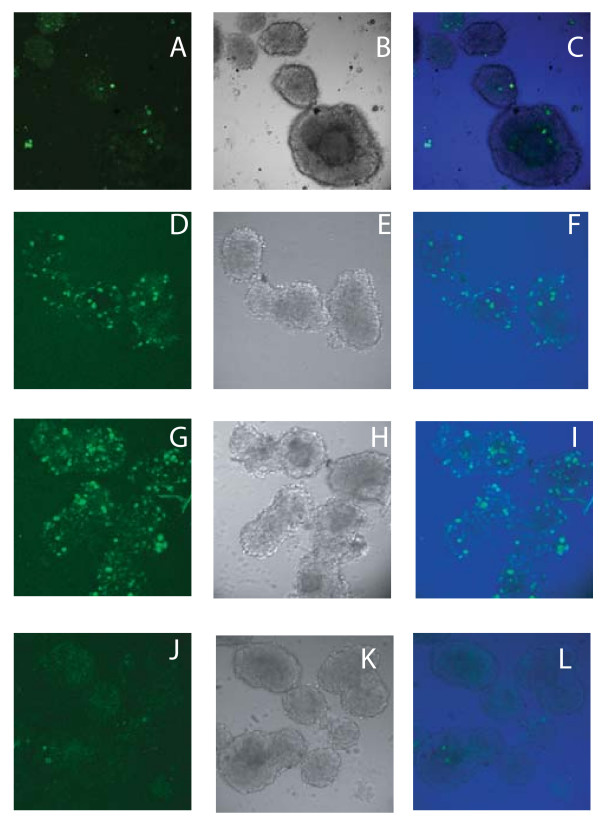
**Transduction of rat islets with pseudotyped AAV vectors and a high Ad5 MOI**. Rat islets were transduced with AAV2-nls-eGFP packaged into AAV2 (A-C), AAV2apoE (D-F), AAV5 (G-I) and AAV8 (J-L) capsids at an AAV MOI of 1500 (A-L). All islets were infected with Ad5 at an MOI of 10. The islets were visualized by confocal microscopy on day 5 after transduction. (A, D, G, J) Fluorescent. (B, E, H, K) Differential interference contrast (DIC). (C, F, I, L) Merged.

**Figure 2:**
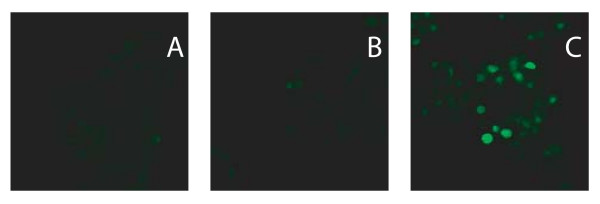
**Transduction of rat islets with a high MOI of pseudotyped AAV2/8 vectors and a high Ad5 MOI**. Rat islets were transduced with AAV2-nls-eGFP packaged into an AAV8 capsid at an AAV MOI of 1500 (B) or 10,000 (C). All islets were infected with Ad5 at an MOI of 10. (A) Ad5 only. The islets were visualized by fluoresence confocal microscopy on day 5 after transduction.

Rat islets (Figs. 1A–L) were transduced with recombinant AAV2 genomes harboring the nls-eGFP gene packaged into AAV2, AAV2apoE, AAV5, or AAV8 capsids at an AAV MOI of 1.5 × 10^3 ^and an Ad5 MOI of 10. At this MOI, AAV2/2 did not transduce rat islets well (Figs. 1A–C). AAV2apoE, however, did show a modest ability to transduce rat islets (Figs. 1D–F). The most efficient vector in this experiment was AAV2/5 (Figs. 1G–I). AAV2/8 appeared to be even less efficient than AAV2/2 at this MOI (Figs. 1J–L). We were surprised at the relative lack of transduction with the AAV2/8 vector under these conditions, given previous reports that it was more efficient at mouse islet transduction than AAV2/2 or AAV2/5 vectors [19,20]. However, increasing the AAV MOI to 1.0 × 10^4^, while maintaining an Ad5 MOI of 10, did facilitate AAV2/8 transduction of rat islets (Fig. 2C).

**Figure 3:**
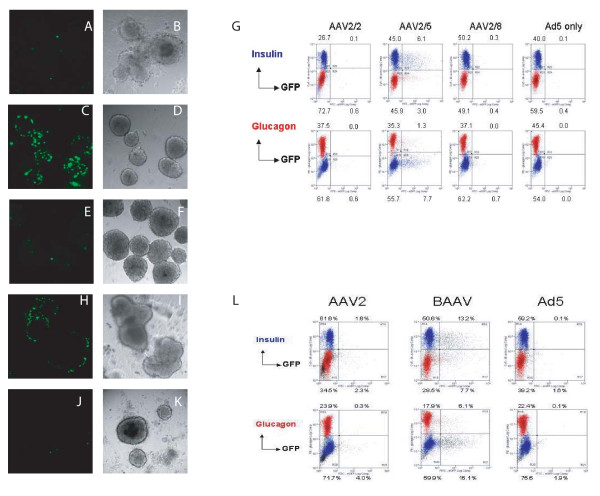
**Transduction of rat islets with pseudotyped AAV vectors at high AAV MOIs and a low Ad5 MOI**. Rat islets were transduced with AAV2-nls-eGFP packaged into the capsids of AAV2 (A, B, J, K), AAV5 (C, D), AAV8 (E, F) or BAAV (H, I), at an AAV MOI of 10,000 (A-G) or 30,000 (H-L) and an Ad5 MOI of 1 (A-L). The islets were visualized by confocal microscopy on day 5 after transduction. (A, C, E, H, J) Fluorescent. (B, D, F, I, K) DIC. On day 6, flow cytometry analysis was performed on dispersed islet cells (G, L), including negative controls infected with Ad5 only. AAV vectors used in panels A-G were purified on iodixanol gradients. AAV vectors used in panels H-L were purified on cesium chloride gradients.

Vectors were also tested at an AAV MOI of 1 × 10^4 ^and an Ad5 MOI of 1 (Figs. 3A–F). AAV2/2 again showed minimal transduction (Figs. 3A–B). AAV2/8 also did not transduce rat islets very well at this MOI (Figs. 3E–F), but significant transduction was achieved with AAV2/5 (Figs. 3C–D), suggesting that the requirement for Ad5 helper functions for AAV2/5 is less than that of AAV2/8.

Previous works have attempted to quantify transduction by microscopy. We used a recently developed method for dispersing islet cells, prior to flow cytometry analysis [21], which showed that 9% of the AAV2/5- treated islet cells were positive for GFP (Fig. 3G). AAV2/5 transduced beta-islet cells, since 6% of the islet cells (12% of insulin^+ ^cells) were doubly-positive for GFP and insulin. Fewer alpha cells were transduced than beta cells, as only 1% of cells were positive for GFP and glucagon (3% of glucagon^+ ^cells).

We also compared rat islets transduced with a recombinant AAV2 genome encoding eGFP packaged into the capsids of AAV2 or BAAV at an AAV MOI of 3.0 × 10^4 ^and an Ad5 MOI of 1 (Figs. 3H–L). The AAV MOI was increased in this experiment over the MOIs in the other experiments, because these viruses were purified using CsCl density gradients while the other viruses had been purified using an iodixanol gradient followed by ion exchange or heparin chromatography. AAV vectors purified by a CsCl gradient exhibit decreased infectivity compared to AAV purified by an iodixanol gradient and affinity chromatography [22].

The CsCl-purified AAV2/2 vector showed little transduction of rat islets (Figs. 3J–L). However, approximately 21% of the BAAV-treated islets cells were transduced (Figs. 3H, I, L). Furthermore, 13% of the islet cells were doubly-positive for insulin and eGFP (21% of insulin-positive cells), suggesting that BAAV can transduce beta-islet cells. Unlike AAV2/5, BAAV appears to be as efficient at transducing alpha cells, since 6% of the islet cells (25% of the glucagon^+ ^cells) were positive for eGFP and glucagon (Fig. 3L).

### Xenotransplantation of AAVTGFP2/5-Transduced Rat Islets

**Figure 4:**
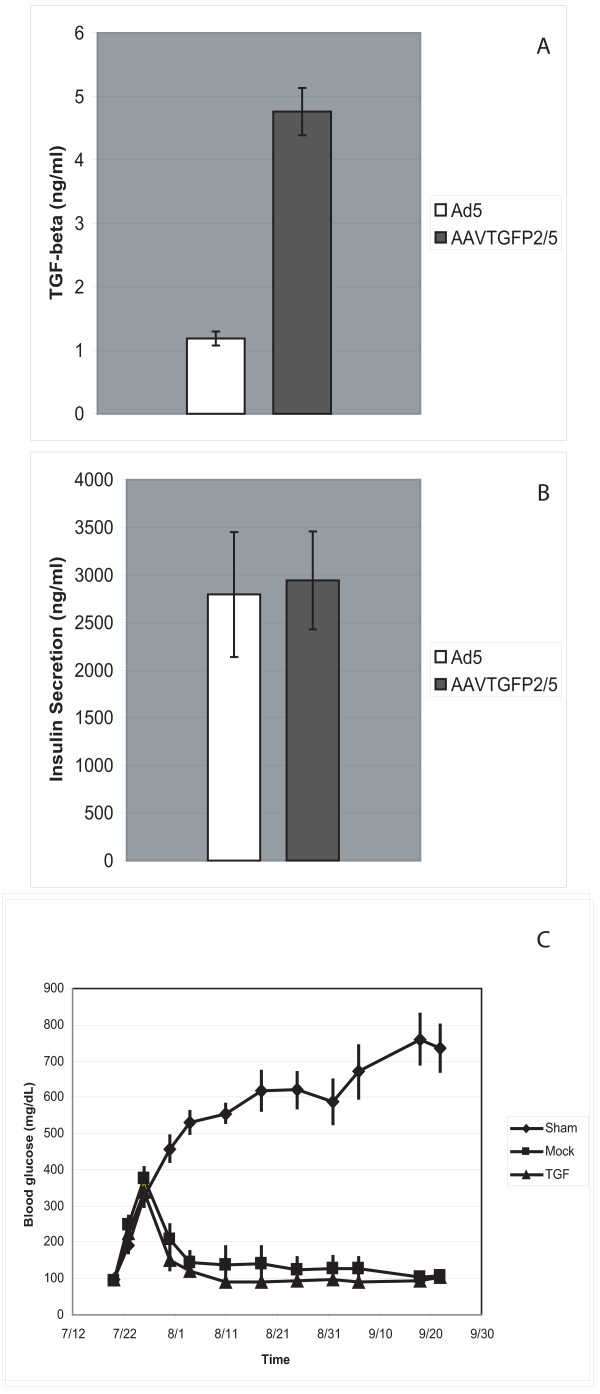
**Transduction of rat islets with AAVTGFP2/5**. (A) *In vitro *expression of TGF-beta 1 in conditioned medium taken on day 6 after transduction of rat islets with AAVTGFP2/5 (plus infection with Ad5) as measured by ELISA. As a negative control, islets were infected with Ad5 only. The error bars indicate the standard deviation. (B) Insulin secretion *in vitro *from rat islets transduced with AAVTGFP2/5 and infected with Ad5. Insulin levels in conditioned medium on day 6 after transduction were measured by radioimmunoassay. As a negative control, rat islets were infected with Ad5 only. The error bars indicate the standard deviation. (C) Blood glucose levels of NOD-SCID mice transplanted with transduced rat islets. Islets were isolated from Wistar rats and transduced with AAVTGFP2/5. Transduced islets were then transplanted into diabetic NOD-SCID mice (TGF). Other NOD-SCID mice were either transplanted with mock-transduced islets or sham operated. The error bars indicate the standard error (n = 6).

We wished to assess the practicality of transducing islets with an AAV vector encoding the immunosuppressive gene, TGF-β1. It is possible that transduction with an AAV- TGF-β1 vector might alter islet function. Rat islets were therefore transduced with an AAV5 pseudotyped vector harboring the gene for TGF-β1, linked via an internal ribosome entry site (IRES) to the eGFP gene (AAVTGFP2/5), at an MOI of 1.0 × 10^4 ^and an Ad5 MOI of 1 *in vitro*. On day 6 after treatment, transduced islets showed a four-fold increase in supernatant TGF-β1 levels compared to control islets infected only with Ad5 (Fig. 4A). Transduced islets had levels of insulin secretion comparable to islets infected with Ad5 alone (Fig. 4B). Finally, rat islets (300 per mouse) were transplanted into female, immune-deficient NOD-SCID mice that had been made diabetic with streptozotocin treatment. The mice became normoglycemic shortly after transplantation of either untreated or transduced (AAVTGFP2/5 without Ad5) islets and remained normoglycemic for more than two months (Fig. 4C). Glucose levels continued rising in mice receiving no islets (Fig. 4C).

## Discussion

Vectors were produced containing recombinant AAV2 genomes, harboring the nls-eGFP gene, packaged into the capsids of AAV2, AAV5, AAV8, BAAV, or an AAV2 capsid that was modified by inserting a polypeptide containing the ApoE receptor binding domain of the ApoE ligand connected to a lipid binding domain [23].

Typically, an Ad5 MOI of 5 has been used for the *in vitro *transduction of islets [12,16], but Ad5 MOIs from 1–50 are able to enhance AAV transduction in proportion to the Ad5 MOI [24]. We tested an Ad5 MOI of 1 to determine if a low concentration of Ad5 could provide helper functions, while perhaps minimizing Ad5 toxicity. At an Ad5 MOI of 1, transduction of rat islets was more efficient with the AAV2/5 vector than with the AAV2/2 or AAV2/8 vectors. The platelet-derived growth factor receptor (PDGFR) is a receptor for AAV5 [25]. PDGFR levels in islets have been reported to vary between human subjects and are particularly high in patients with chronic pancreatitis [26].

Given the unexpectedly poor transduction efficiency of the AAV2/8 vector, it was deemed necessary to test it in the presence of a higher Ad5 MOI. Increasing the Ad5 MOI to 10 significantly enhanced AAV2/8 transduction of rat islets.

Although Ad5 can enhance AAV2 transduction by stimulating AAV2 DNA second-strand synthesis [24,27], Ad5 capsids also facilitate translocation of AAV2 into the nucleus [28]. Since our AAV2/5 and AAV2/8 vectors have identical DNA templates, the lower Ad5 MOI is probably sufficient for second-strand synthesis. Therefore, the improved transduction of AAV2/8 with an Ad5 MOI of 10 may be due to enhanced nuclear translocation or viral uncoating. Please note that *in vivo *experiments with AAV vectors are usually done in the absence of helper virus, therefore, relative gene expression *in vivo *may differ from that seen *in vitro*. However, the lower Ad5 MOI should more closely approximate *in vivo *conditions without helper virus.

It has been reported that AAV2/5 vectors can transduce human islets [16]. This increases the probability that data obtained with AAV2/5 vectors in rat to mouse transplantations can be translated into patient protocols.

BAAV transduces rat islets relatively well at an Ad5 MOI of 1. However, since BAAV transduced α-islet cells with about the same efficiency as β-islet cells, the AAV5 capsid may be better for more selective transduction of β-islet cells.

Our results suggest that AAVTGFP2/5, encoding TGF-β1, can transduce islets without altering islet functions *in vitro *or *in vivo*. These data concur with a previous experiment in which adenovirus vector delivery of TGF-β1 to islets showed no impact on insulin production, in response to glucose, in culture [9]. The amount of insulin contributed by the serum used to prepare the culture medium was small (< 0.1 ng/ml final concentration) compared to the amount produced by the islets. An immunodeficient diabetic mouse model was chosen for transplantation to allow better analysis of direct toxicity from TGF-β1. Our mice receiving AAVTGFP2/5-transduced islets became normoglycemic for at least 2 months. Previous studies of AAV vectors with islets showed transgene expression within 2–4 weeks [12,15]. We therefore assume that the TGF-β1 gene was expressed for at least 4 weeks. We had hoped that the eGFP gene within AAVTGFP2/5 would allow us to confirm gene expression, but the eGFP signal was too weak to detect reliably, even under tissue culture conditions (not shown).

## Conclusion

Infection of rat islets with AAV2/5 vectors allows relatively efficient transgene expression with no significant adverse effects on *in vivo *islet function. TGF-β1, expressed from an AAV2/5 vector, does not appear to have a negative impact on rat islet function. AAV2/5 vectors may therefore be useful for pre-treating donor islets prior to transplantation.

## Methods

### Cell Culture

Packaging of AAV2 genomes into AAV2, AAV2apoE, AAV5 and AAV8 capsids used HEK 293 cells (Stratagene; La Jolla, CA). Cells were maintained at 37°C with a humidified environment containing 5% CO_2_, in Dulbecco's Modified Eagle Medium (Invitrogen; Carlsbad, CA) with 10% fetal bovine serum (FBS) (Invitrogen).

### Packaging of AAV Vectors

Plasmid pAAV2-nls-EGFP contains the enhanced green fluorescent protein gene, modified to contain a nuclear localization sequence (nls-eGFP), controlled by the human cytomegalovirus major immediate-early promoter, flanked by the AAV2 inverted terminal repeats (ITRs). At 24 h before transfection, twenty 15-cm diameter plates were each seeded with 1.7 × 10^7 ^HEK 293 cells. For transfection, 360 μg of pAAV2-nls-EGFP, 1.08 mg of pHelper (Stratagene), and 360 μg of either pAAV-RC (Stratagene), pAAV2apoE (described below), p5E18-VD2/8 [29] (from Dr. James Wilson, University of Pennsylvania, USA), or pXR5 [30] (from Dr. R. Jude Samulski, University of North Carolina, USA) were added to 25 ml of 0.3 M CaCl_2_. Plasmids pAAV-RC, p5E18-VD2/8 and pXR5 contain AAV genes necessary for packaging AAV2 genomes into AAV2, AAV8 and AAV5 capsids, respectively, and pHelper contains adenovirus 5 (Ad5) genes required for helper functions. Twenty-five milliliters of 2× HBS (280 mM NaCl, 1.5 mM Na_2 _PO_4_, 50 mM HEPES, pH 7.05), prewarmed to 37°C, was added to the plasmid/CaCl_2 _solution, and the mixture was incubated for 1 minute at room temperature. Next, this mixture was added to 400 ml of pre-warmed DMEM with 10% FBS and 22 ml of this medium was added to each 15-cm diameter plate. Virus was purified by the method of Zolotukhin, et al. [22,31]. In brief, after 48 hours, the cells were harvested by centrifugation at 1140 × g for 10 minutes and the supernatant was discarded. The virus was released by three freeze-thaw cycles, then purified on an iodixanol gradient, followed by either heparin affinity (AAV2 and AAV2apoE) or ion exchange (AAV2/5 and AAV2/8) chromatography.

The BAAV-eGFP vector was produced and purified using a cesium chloride gradient, as described previously [32]. A batch of AAV2/2-eGFP vector, used for comparison, was purified by the same method.

### Construction of pAAV2apoE

We inserted the ApoE ligand into the AAV2 capsid using overlapping PCR mutagenesis to introduce AscI and PacI restriction sites for the cloning of the DNA encoding the ligand into the capsid gene. The mutagenic primers were 5'ggcgcgccttaattaacgtcttaacaggttcc3' (M1) and 5'ttaattaaggcgcgccgctccgggaaaaaagaggc' (M2). PCR was performed with two separate reactions. In reaction 1, pAAVRC was used as a template and primers M1 and 5'ggacgtacgggagctggtacttccg3' were used for amplification. In reaction 2, the same template and primers M2 and 5'gatttaaatcaggtatggctgccg3' were used. The PCR fragments from these reactions were gel purified, annealed to each other, and extended with pfu Ultra DNA polymerase (Stratagene). The resulting fragment was cut with BsiWI and SwaI, and ligated into the corresponding sites of pAAVRC to make pAAVRCKI. Next, oligonucleotides containing the sequence for the ApoE ligand (LRKLRKRLLRDWLKAFYDKVAEKLKEAF) (synthesized by Integrated DNA Technologies; Coralville, Iowa, USA), flanked by AscI and PacI sites, were annealed, cut with AscI and PacI, and inserted into the corresponding sites of pAAVRCKI to make pAAV2apoE. Loiler *et*. *al*. reported improved transduction of islets with an AAV2 vector by insertion of a similar peptide [13]. Our peptide was modified by substituting lysine residues for aspartic acid residues at positions 23 and 25, since the lipid binding domain is a class A amphipathic α-helix, which is characterized by positively charged residues at these positions [33].

### Construction of pAAVTGFP

The rat TGF-β1 gene was PCR amplified from plasmid TGFβ33 (from Dr. Anita Roberts, National Cancer Institute) and cloned into pEGFPIRES (Clontech; Mountain View, California, USA), which contains an eGFP gene downstream of an internal ribosome entry site (IRES). The primers that amplified the TGF-β1 gene introduced an EcoRV site on the 5' end of the gene and an Eco RI site on the 3' end. The 5' primer was 5'attcgatatcggcgccgcctcccccatgccg3' and the 3' primer was 5'attcgaattccggggcctcagctgcacttgcagg3'. The PCR parameters were: 2 minutes at 95°C; then 30 cycles of 95°C for 30 seconds, 55°C for 30 seconds, and 72°C for 1 minute and 12 seconds; then a final 10 minute extension period at 72°C.

After gel purification, the TGF-β1 fragment and pEGFPIRES were digested with EcoRI and EcoRV (New England Biolabs; Ipswich, Massachusetts, USA) and ligated to form pTGFP. Next, the expression cassette from pTGFP, containing the TGF-β1 and eGFP genes, was amplified by PCR using the primers 5'attagcggccgccgggccagatatacgcgttga3' and 5'attagcggccgctcttacgtgagctcggggtc3', which included NotI sites. PCR conditions were the same as above, with pTGFP as the template. The PCR product was digested with NotI and ligated to the NotI fragment of pAAVLacZ (Stratagene) containing the AAV2 ITRs, creating pAAVTGFP. Packaging of pAAVTGFP into the AAV5 capsid was then done using the method described above.

### Determination of viral titers

For titration of packaged virus genomes, DNA was isolated from 27.8 μl of the final virus solution. The volume was adjusted to 100 μl with 10 mM Tris-HCl [pH 7.5], 1 mM EDTA. Next, 10 μl of DNase buffer (500 mM Tris-HCL, pH 7.5; 100 mM MgCl_2_), and 10 U of DNase I (Roche Applied Science; Indianapolis, Indiana, USA) was added and the solution was incubated at 37°C for 1 hr. Next, 10 μl of proteinase K buffer (100 mM Tris-HCl, pH 8.0; 100 mM EDTA; 10% SDS) was added with 18.6 μg of proteinase K (Invitrogen). The solution was incubated for 1 hr at 37°C. Proteins were removed by two phenol-chloroform extractions and one chloroform extraction. The DNA was ethanol precipitated with 10 μg of glycogen (Roche). The DNA was resuspended in water and dot blot hybridization was performed using linearized pAAV2-nls-EGFP as standards.

Adenovirus type 5 was a kind gift from Dr. Irving Miller (National Institutes of Health). It was purified on cesium chloride gradients and titered by plaque assays as described previously [34].

### Isolation of Rat Islets

Wistar rats were euthanized by CO_2 _inhalation. Next, 10 ml of a 0.25 mg/ml solution of Liberase RI (Roche) in DMEM was injected into the common bile duct. Each pancreas was removed, placed in a 50 ml tube and incubated at 37°C for 25 min. After the incubation, 40 ml of cold DMEM + 10% FBS was added. Tubes were shaken vigorously for 5–10 sec by hand to break up the tissue. The rest of the isolation was done at room temperature. Tubes were centrifuged at 1000 rpm for 1 min, the supernatant was poured off, 35 ml of DMEM (without serum) was added and vortexed gently. Centrifugation was repeated and the supernatant was discarded. The tissue was resuspended in 10 ml of DMEM and filtered through a wire mesh with 1.5 mm holes to remove the remaining undigested tissue, fat and lymph. An additional 5 ml of DMEM was added to the original tube to wash out any remaining islets and the wash was also filtered through the wire mesh. This filtrate was then filtered through a wire mesh with 0.8 mm holes, then centrifuged at 1200 rpm for 90 sec. The supernatant was aspirated and the pellet was resuspended in 20 ml of Ficoll and overlain with 10 ml of DMEM. The sample was spun for 15 min at 1900 rpm. The topmost layer of media was aspirated. The islet layer was then collected from the interface with a 10 ml pipette and placed in a new 50 ml tube. The islets were washed several times with DMEM and resuspended in 10 ml of DMEM. Islets were hand picked for later procedures.

### Islet Transductions

All islet transductions were performed at least in duplicate and were done on the day of isolation. The multiplicity of infection (MOI) was based on an average of 1000 cells per islet. Fifty islets per well were placed in a 24-well, glass-bottomed, tissue culture plate with 13 mm diameter microwells (MatTek Corp.; Ashland, Massachusetts, USA) in CMRL-1066 medium (Invitrogen) with 10% FBS. The volumes of the viral preps were adjusted with Lactated Ringer's Solution before being suspended in CMRL (without FBS) at a final volume of 300 μl. The medium was aspirated from the islets, and the islets were resuspended in the viral suspensions. Islets were then incubated at 37°C for two hours. Following incubation, 700 μl of CMRL with 10% FBS was added per well.

On day 5 after transduction the islets were viewed on an LSM410 laser scanning confocal microscope (Carl Zeiss; Thornwood, New York, USA) with a 488/568 krypton-argon omnichrome laser (Melles Griot; Carlsbad, California, USA). Individual optical sections of GFP-expressing islets were collected at 1 micron optical thickness using 488 nm excitation, a 20× neofluar lens at zoom 2.5 and a BP 515–540 nm emission filter. In some cases 1 micron optical sections were collected at every 0.5 micron focus step through the islet and reconstructed into a maximum projection of the entire islet by LSM410 software.

On day 6 after transduction the islets were dispersed into single cells by incubation in PBS buffer (without Mg^++ ^or Ca^++^) for 10 minutes at room temperature. The percentages of GFP^+^, insulin^+^, and glucagon^+ ^cells were determined by flow cytometry, as described previously [21,35].

Insulin in conditioned medium from islet cultures was detected using a radio immunoassay kit (Linco Research, Inc.; St. Charles, Missouri). TGF-β1 in conditioned medium was detected with an enzyme-linked immunosorbent assay (ELISA) kit (Alpco diagnostics, Salem, New Hampshire, USA).

### Transplantation of Transduced Islets into NOD-SCID mice

To ensure that mice became diabetic at approximately the same time, female NOD-SCID mice were treated with 40 mg/kg streptozotocin (Sigma) freshly dissolved in citrate buffer (pH 4.5), injected intraperitoneally once a day for 4–5 days. Blood glucose was measured daily using a Glucometer Elite XL (Bayer Corp. Elkhart, Indiana, USA). At day 7, after the mice became diabetic (blood glucose of 250–450 mg/dl), 300 rat islets, that were transduced with AAVTGFP2/5 at an MOI of 15,000 (without helper virus), were transplanted under the kidney capsule of 6 mice. As negative controls, 6 mice were transplanted with mock-transduced islets, and 6 mice were opened by a dorsal incision and closed without receiving islets (Sham operated). Blood samples were collected at various times for glucose testing. All *in vivo *procedures were approved by the NIDDK animal care and use committee.

## Competing interests

R.A.O. is a co-inventor on several patents involving AAV vectors. To the extent that this work will increase the value of those patents, he has a competing interest.

## Authors' contributions

AC was the primary contributor to project conception, overall experimental design, plasmid construction, virus production, islet infection, data analysis and writing of manuscript. OG isolated islets, designed and supervised transplant experiments, and contributed to writing of the manuscript. ND performed microscopy and contributed to data analysis and writing of the manuscript. WJ performed islet transplantations, TGF-beta and insulin assays, blood glucose monitoring and contributed to data analysis and writing of the manuscript. SP assisted with islet isolation, islet transplantation and blood glucose monitoring. EL performed islet isolation and assisted with experimental design and writing of the manuscript. KP designed, performed and analyzed flow cytometry and contributed to writing of the manuscript. MS prepared and titered cesium chloride-purified virus and contributed to writing of the manuscript. VMA designed and constructed plasmids and assisted with data analysis and preparation of the manuscript. JC supervised preparation of cesium chloride-purified virus and contributed to writing of the manuscript. EBM supervised microscopy. DH assisted with experimental design, data analysis and preparation of the manuscript. RO was overall project coordinator, and contributed to experimental design, data analysis and writing of the manuscript.
